# Global Dynamics of Certain Homogeneous Second-Order Quadratic Fractional Difference Equation

**DOI:** 10.1155/2013/210846

**Published:** 2013-12-04

**Authors:** M. Garić-Demirović, M. R. S. Kulenović, M. Nurkanović

**Affiliations:** ^1^Department of Mathematics, University of Tuzla, 75000 Tuzla, Bosnia and Herzegovina; ^2^Department of Mathematics, University of Rhode Island, Kingston, RI 02881-0816, USA

## Abstract

We investigate the basins of attraction of equilibrium points and minimal period-two solutions of the difference equation of the form *x*_*n*+1_ = *x*_*n*−1_^2^/(*ax*_*n*_^2^ + *bx*_*n*_ *x*_*n*−1_ + *cx*_*n*−1_^2^), *n* = 0,1, 2,…, where the parameters *a*,  *b*, and  *c* are positive numbers and the initial conditions *x*_−1_ and *x*_0_ are arbitrary nonnegative numbers. The unique feature of this equation is the coexistence of an equilibrium solution and the minimal period-two solution both of which are locally asymptotically stable.

## 1. Introduction

We investigate global behavior of the equation

(1) $$\begin{array}{c} x_{n+1}=\frac{x_{n-1}^{2}}{ax_{n}^{2}+bx_{n}x_{n-1}+cx_{n-1}^{2}}=f\left(x_{n},x_{n-1}\right), \end{array}$$

where the parameters  *a*,  *b*, and  *c*  are positive numbers and the initial conditions  *x*_−1_,   and *x*_0_  are arbitrary nonnegative numbers such that  *x*_−1_ + *x*_0_ > 0. Equation (1) is a special case of equations

(2) $$\begin{array}{c} x_{n+1}=\frac{Ax_{n}^{2}+Bx_{n}x_{n-1}+Cx_{n-1}^{2}}{ax_{n}^{2}+bx_{n}x_{n-1}+cx_{n-1}^{2}},\;n=0,1,2,\ldots , \end{array}$$

(3)  xn+1=Axn2+Bxnxn−1+Cxn−12+Dxn+Exn−1+Faxn2+bxnxn−1+cxn−12+dxn+exn−1+f,n=0,1,2,….

Some special cases of (3) have been considered in the series of papers [1–5]. Some special second-order quadratic fractional difference equations have appeared in analysis of competitive and anticompetitive systems of linear fractional difference equations in the plane; see [6–10]. Describing the global dynamics of (3) is a formidable task as this equation contains as a special case many equations with complicated dynamics, such as the linear fractional difference equation

(4) $$\begin{array}{c} x_{n+1}=\frac{Dx_{n}+Ex_{n-1}+F}{dx_{n}+ex_{n-1}+f},\;n=0,1,2,\ldots \end{array}$$

which dynamics was investigated in great detail in [11] and in many papers which solved some conjectures and open problems posed in [11]. Equation (2) can be brought to the form

(5) $$\begin{array}{c} x_{n+1}=\frac{A{\left(x_{n}/x_{n-1}\right)}^{2}+B\left(x_{n}/x_{n-1}\right)+C}{a{\left(x_{n}/x_{n-1}\right)}^{2}+b\left(x_{n}/x_{n-1}\right)+c},\;n=0,1,2,\ldots , \end{array}$$

and one can take the advantage of this auxiliary equation to describe the dynamics of (2). This approach was used in [1–4, 12]. In this paper, we take a different approach based on the theory of monotone maps developed in [13, 14] and use it to describe precisely the basins of attraction of all attractors of this equation. The special case of (1) when  *a* = 0  is the linear fractional difference equation whose global dynamics is described in [11]. We show that (1) exhibits three types of global behavior characterized by the existence of a unique positive equilibrium solution and one or two minimal period-two solutions, one of which is locally stable and the other is a saddle point. The unique feature of (1) is the coexistence of an equilibrium solution and the minimal period-two solution both being locally asymptotically stable. This new phenomenon is caused by the presence of quadratic terms and did not exist in the case of (4).

Our results will be based on the following theorem for a general second-order difference equation

(6) $$\begin{array}{c} x_{n+1}=f\left(x_{n},x_{n-1}\right),\;n=0,1,2,\ldots ; \end{array}$$

see [15].

**Theorem 1**

Let  *I*  be a set of real numbers and let  *f* : *I* × *I* → *I*  be a function which is nonincreasing in the first variable and nondecreasing in the second variable. Then, for every solution  {*x*_*n*_}_*n*=−1_^*∞*^  of the equation

(7) $$\begin{array}{c} x_{n+1}=f\left(x_{n},x_{n-1}\right),\;x_{-1},x_{0}\in I,\;\;n=0,1,2,\ldots , \end{array}$$

the subsequences  {*x*_2*n*_}_*n*=0_^*∞*^  and  {*x*_2*n*−1_}_*n*=0_^*∞*^  of even and odd terms of the solution do exactly one of the following:

- eventually, they are both monotonically increasing;
- eventually, they are both monotonically decreasing;
- one of them is monotonically increasing and the other is monotonically decreasing.

The consequence of Theorem 1 is that every bounded solution of (7) converges to either equilibrium or period-two solution or to the point on the boundary, and the most important question becomes determining the basins of attraction of these solutions as well as the unbounded solutions. The answer to this question follows from an application of theory of monotone maps in the plane which will be presented in Section 2.

## 2. Preliminaries

We now give some basic notions about monotone maps in the plane.

Consider a partial ordering  ⪯  on  ℝ^2^. Two points  *x*, *y* ∈ ℝ^2^  are said to be related if  *x*⪯*y*  or  *y*⪯*x*. Also, a strict inequality between points may be defined as  *x*≺*y*  if  *x*≺*y*  and  *x* ≠ *y*. A stronger inequality may be defined as  *x* = (*x*_1_, *x*_2_) ≪ *y* = (*y*_1_, *y*_2_)  if  *x*⪯*y*  with  *x*_1_ ≠ *y*_1_  and  *x*_2_ ≠ *y*_2_.

A map *T* on a nonempty set *ℛ* ⊂ ℝ^2^ is a continuous function *T* : *ℛ* → *ℛ*. The map *T* is monotone if *x*⪯*y* implies that *T*(*x*)⪯*T*(*y*) for all *x*, *y* ∈ *ℛ*, and it is strongly monotone on *ℛ* if *x*≺*y* implies that *T*(*x*) ≪ *T*(*y*) for all *x*, *y* ∈ *ℛ*. The map is strictly monotone on *ℛ* if *x*≺*y* implies that *T*(*x*)≺*T*(*y*) for all *x*, *y* ∈ *ℛ*. Clearly, being related is invariant under iteration of a strongly monotone map.

Throughout this paper, we will use the *North-East ordering* (NE) for which the positive cone is the first quadrant; that is, this partial ordering is defined by  (*x*_1_, *y*_1_) ⪯_ne_(*x*_2_, *y*_2_)  if  *x*_1_ ≤ *x*_2_  and  *y*_1_ ≤ *y*_2_  and the South-East (SE) ordering defined as  (*x*_1_, *y*_1_) ⪯_se_(*x*_2_, *y*_2_)  if  *x*_1_ ≤ *x*_2_  and  *y*_1_ ≥ *y*_2_.

A map *T* on a nonempty set *ℛ* ⊂ ℝ^2^ which is monotone with respect to the North-East ordering is called *cooperative* and a map monotone with respect to the South-East ordering is called *competitive*.

If *T* is a differentiable map on a nonempty set *ℛ*, a sufficient condition for *T* to be strongly monotone with respect to the SE ordering is that the Jacobian matrix at all points *x* has the following sign configuration:

(8) $$\begin{array}{c} \;\mathrm{sign}\left(J_{T}\left(\text{x}\right)\right)=\left[\begin{array}{cc} + & -\\ - & + \end{array}\right], \end{array}$$

provided that *ℛ* is open and convex.

For *x* ∈ ℝ^2^, define *Q*_*ℓ*_(*x*) for *ℓ* = 1,…, 4 to be the usual four quadrants based at *x* and numbered in a counterclockwise direction; for example, *Q*_1_(*x*) = {*y* ∈ *R* ^2^ : *x*_1_ ≤ *y*_1_, *x*_2_ ≤ *y*_2_}. Basin of attraction of a fixed point $\left(\overset{-}{x},\overset{-}{y}\right)$ of a map *T*, denoted as $ℬ((\overset{-}{x},\overset{-}{y}))$ , is defined as the set of all initial points (*x*_0_, *y*_0_) for which the sequence of iterates *T* ^*n*^((*x*_0_, *y*_0_)) converges to $\left(\overset{-}{x},\overset{-}{y}\right)$ . Similarly, we define a basin of attraction of a periodic point of period *p*. The next five results, from [13, 14], are useful for determining basins of attraction of fixed points of competitive maps. Related results have been obtained by Smith in [5, 16].

**Theorem 2**

Let  *T*  be a competitive map on a rectangular region *ℛ* ⊂ ℝ^2^. Let $\bar{x}\in ℛ$ be a fixed point of  *T*  such that $\;\Delta :=ℛ\cap \mathrm{int}(Q_{1}(\bar{x})\cup Q_{3}(\bar{x}))\;$ is nonempty (i.e., $\;\bar{x}\;$ is not the NW or SE vertex of *ℛ*), and  *T*  is strongly competitive on  Δ. Suppose that the following statements are true.

- The map  *T*  has a  *C* ^1^  extension to a neighborhood of $\bar{x}$ .
- The Jacobian $J_{T}(\bar{x})$ of *T* at $\bar{x}$ has real eigenvalues *λ*, *μ* such that 0 < |*λ* | <*μ*, where |*λ* | <1, and the eigenspace *E* ^*λ*^ associated with *λ* is not a coordinate axis.

Then, there exists a curve *𝒞* ⊂ *ℛ* through $\bar{x}$ that is invariant and a subset of the basin of attraction of $\bar{x}$ , such that *𝒞* is tangential to the eigenspace *E* ^*λ*^ at $\bar{x}$ , and *𝒞* is the graph of a strictly increasing continuous function of the first coordinate on an interval. Any endpoints of *𝒞* in the interior of *ℛ* are either fixed points or minimal period-two points. In the latter case, the set of endpoints of *𝒞* is a minimal period-two orbit of *T*.

We will see in Theorem 4 that the situation where the endpoints of *𝒞* are boundary points of *ℛ* is of interest. The following result gives a sufficient condition for this case.

**Theorem 3**

For the curve *𝒞* of Theorem 2 to have endpoints in  ∂*ℛ*, it is sufficient that at least one of the following conditions is satisfied.

- The map  *T*  has no fixed points nor periodic points of minimal period-two in  Δ.
- The map  *T*  has no fixed points in  Δ, $\;\det J_{T}(\bar{x})>0$ , and $\;T(x)=\bar{x}\;$ has no solutions  *x* ∈ Δ.
- The map  *T*  has no points of minimal period-two in  Δ, $\;\det J_{T}(\bar{x})<0$ , and $\;T(x)=\bar{x}\;$ has no solutions  *x* ∈ Δ.

For maps that are strongly competitive near the fixed point, hypothesis (b) of Theorem 2 reduces just to | *λ* | <1. This follows from a change of variables [5] that allows the Perron-Frobenius theorem to be applied. Also, one can show that in such case no associated eigenvector is aligned with a coordinate axis.

The next result is useful for determining basins of attraction of fixed points of competitive maps.

**Theorem 4**

(A) Assume the hypotheses of Theorem 2, and let *𝒞* be the curve whose existence is guaranteed by Theorem 2. If the endpoints of *𝒞* belong to ∂*ℛ*, then *𝒞* separates *ℛ* into two connected components, namely,

(9) $$\begin{array}{c} {𝒲}_{-}:=\left\{x\in ℛ\setminus 𝒞:\exists y\in 𝒞\;with\;\text{ x }{⪯}_{\text{se}}\;y\right\},\\ {𝒲}_{+}:=\left\{x\in ℛ\setminus 𝒞:\exists y\in 𝒞\;with\;y\;{⪯}_{\text{se}}\;x\right\}, \end{array}$$

such that the following statements are true.

-   
*𝒲*_−_  is invariant, and $\;\mathrm{dist}(T^{n}(x),Q_{2}(\bar{x}))\to 0\;$ as  *n* → *∞*  for every  *x* ∈ *𝒲*_−_.
-   
*𝒲*_+_  is invariant, and $\;\mathrm{dist}(T^{n}(x),Q_{4}(\bar{x}))\to 0\;$ as  *n* → *∞*  for every  *x* ∈ *𝒲*_+_.

(B) If, in addition to the hypotheses of part (A), $\;\bar{x}\;$ is an interior point of *ℛ* and  *T*  is  *C* ^2^  and strongly competitive in a neighborhood of $\;\bar{\text{x}}$ , then  *T*  has no periodic points in the boundary of $\;Q_{1}(\bar{x})\cup Q_{3}(\bar{x})\;$ except for $\;\bar{x}$ , and the following statements are true.

(iii) For every  *x* ∈ *𝒲*_−_, there exists  *n*_0_ ∈ *ℕ*such that $\;T^{n}(x)\in \mathrm{int}Q_{2}(\bar{x})\;$ for  *n* ≥ *n*_0_.
(iv) For every  *x* ∈ *𝒲*_+_, there exists  *n*_0_ ∈ *ℕ*such that $\;T^{n}(x)\in \mathrm{int}Q_{4}(\bar{x})\;$ for  *n* ≥ *n*_0_.

If  *T*  is a map on a set *ℛ* and if $\;\bar{\text{x}}\;$ is a fixed point of  *T*, the stable set $\;{𝒲}^{s}(\bar{\text{x}})\;$ of $\;\bar{\text{x}}\;$ is the set $\;\{x\in ℛ:T^{n}(x)\to \bar{\text{x}}\}\;$ and unstable set $\;{𝒲}^{u}(\bar{\text{x}})\;$ of $\;\bar{\text{x}}\;$ is the set

(10) {x∈ℛ:there  exists  {xn}n=−∞0⊂ℛ  s.t.  T(xn)=xn+1,  x0=x, lim⁡n→−∞xn=x¯}.

When  *T*  is noninvertible, the set $\;{𝒲}^{s}(\bar{\text{x}})\;$ may not be connected and made up of infinitely many curves or $\;{𝒲}^{u}(\bar{\text{x}})\;$ may not be a manifold. The following result gives a description of the stable and unstable sets of a saddle point of a competitive map. If the map is a diffeomorphism on *ℛ*, the sets $\;{𝒲}^{s}(\bar{\text{x}})\;$ and $\;{𝒲}^{u}(\bar{\text{x}})\;$ are the stable and unstable manifolds of $\;\bar{x}$ .

**Theorem 5**

In addition to the hypotheses of part (B) of Theorem 4, suppose that *μ* > 1 and that the eigenspace *E* ^*μ*^ associated with *μ* is not a coordinate axis. If the curve *𝒞* of Theorem 2 has endpoints in ∂*ℛ*, then *𝒞* is the stable set ${𝒲}^{s}(\bar{x})$ of $\bar{x}$ , and the unstable set ${𝒲}^{u}(\bar{x})$ of $\bar{x}$ is a curve in *ℛ* that is tangential to *E* ^*μ*^ at $\bar{x}$ and such that it is the graph of a strictly decreasing function of the first coordinate on an interval. Any endpoints of ${𝒲}^{u}(\bar{x})$ in *ℛ* are fixed points of *T*.

**Remark 6**

We say that  *f*(*u*, *v*)  is strongly decreasing in the first argument and strongly increasing in the second argument if it is differentiable and has first partial derivative  *D*_1_ *f*  negative and first partial derivative  *D*_2_ *f*  positive in a considered set. The connection between the theory of monotone maps and the asymptotic behavior of (7) follows from the fact that if  *f*  is strongly decreasing in the first argument and strongly increasing in the second argument, then the second iterate of a map associated to (7) is a strictly competitive map on  *I* × *I*; see [14].

Set  *x*_*n*−1_ = *u*_*n*_  and  *x*_*n*_ = *v*_*n*_  in (7) to obtain the equivalent system

(11) $$\begin{array}{c} \begin{array}{c} u_{n+1}=v_{n}\\ v_{n+1}=f\left(v_{n},u_{n}\right), \end{array}\;n=0,1,\ldots . \end{array}$$

Let  *T*(*u*, *v*) = (*v*, *f*(*v*, *u*)). The second iterate  *T* ^2^  is given by

(12) $$\begin{array}{c} T^{2}\left(u,v\right)=\left(f\left(v,u\right),f\left(f\left(v,u\right),v\right)\right) \end{array}$$

and it is strictly competitive on  *I* × *I*  (see [14]).

**Remark 7**

The characteristic equation of (7) at an equilibrium point $\;(\overset{-}{x},\overset{-}{x})$ ,

(13) $$\begin{array}{c} \lambda^{2}-D_{1}f\left(\overset{-}{x},\overset{-}{x}\right)\lambda -D_{2}f\left(\overset{-}{x},\overset{-}{x}\right)=0, \end{array}$$

has two real roots  *λ*, *μ*  which satisfy  *λ* < 0 < *μ*  and | *λ* | <*μ*, whenever  *f*  is strictly decreasing in first and increasing in second variable. Thus, the applicability of Theorems 2–5 depends on the nonexistence of minimal period-two solution.

There are several global attractivity results for (7). Some of these results give the sufficient conditions for all solutions to approach a unique equilibrium and they were used efficiently in [11]. The next result is from [17].

**Theorem 8 (see [17])**

Consider (7) where  *f* : *I* × *I* → *I*  is a continuous function and  *f*  is decreasing in the first argument and increasing in the second argument. Assume that $\;\bar{x}\;$ is a unique equilibrium point which is locally asymptotically stable and assume that  (*φ*, *ψ*)  and  (*ψ*, *φ*)  are minimal period-two solutions which are saddle points such that

(14) $$\begin{array}{c} \left(\varphi ,\psi \right){⪯}_{\text{se}}\left(\bar{x},\bar{x}\right){⪯}_{\text{se}}\left(\psi ,\varphi \right). \end{array}$$

Then, the basin of attraction $ℬ\left(\left(\bar{x},\bar{x}\right)\right)$ of $\left(\bar{x},\bar{x}\right)$ is the region between the global stable sets *𝒲* ^*s*^((*φ*, *ψ*)) and *𝒲* ^*s*^((*ψ*, *φ*)). More precisely,

(15) ℬ((x¯,x¯))={(x,y):∃yu,yl:yu<y<yl, (x,yl)∈𝒲s((φ,ψ)),(x,yu)∈𝒲s((ψ,φ))}.

The basins of attraction *ℬ*((*φ*, *ψ*)) = *𝒲* ^*s*^((*φ*, *ψ*)) and *ℬ*((*ψ*, *φ*)) = *𝒲* ^*s*^((*ψ*, *φ*)) are exactly the global stable sets of (*φ*, *ψ*) and (*ψ*, *φ*).

If  (*x*_−1_, *x*_0_) ∈ *𝒲*_+_((*ψ*, *φ*))  or  (*x*_−1_, *x*_0_) ∈ *𝒲*_−_((*φ*, *ψ*)), then  *T* ^*n*^((*x*_−1_, *x*_0_))  converges to the other equilibrium point or to the other minimal period-two solutions or to the boundary of the region  *I* × *I*.

## 3. Local Stability Analysis

Denote

(16) $$\begin{array}{c} f\left(u,v\right)=\frac{v^{2}}{au^{2}+buv+cv^{2}}, \end{array}$$

and notice that the function  *f*(*u*, *v*)  is decreasing in the first variable and increasing in the second variable. By Theorem 1, for every solutions  {*x*_*n*_}_*n*=−1_^*∞*^  of (1) the subsequences  {*x*_2*n*_}_*n*=0_^*∞*^  and  {*x*_2*n*−1_}_*n*=0_^*∞*^  are eventually monotonic.

It is clear that (1) has a unique positive equilibrium solution $\;\bar{x}=1/(a+b+c)\;$ and that a linearization of (1) is of the form

(17) $$\begin{array}{c} y_{n+1}=sy_{n}+ty_{n-1}, \end{array}$$

where

(18) s=−t=∂f∂u(x¯,x¯)=(−v2(2au+bv)(au2+buv+cv2)2)(x¯,x¯)=−x¯2(2ax¯+bx¯)(ax¯2+bx¯2+cx¯2)2=−1x¯·2a+b(a+b+c)2=−2a+ba+b+c.

**Lemma 9**

Equation (1) has a unique positive equilibrium solution $\;\bar{x}=1/(a+b+c)$ .

- If  3*a* + *b* − *c* < 0, then equilibrium solution $\;\bar{x}\;$ is locally asymptotically stable.
- If  3*a* + *b* − *c* > 0, then equilibrium solution $\;\bar{x}\;$ is a saddle point.
- If  3*a* + *b* − *c* = 0, then equilibrium solution $\;\bar{x}\;$ is nonhyperbolic (with eigenvalues  *λ*_1_ = −1  and  *λ*_2_ = 1/2).

**Proof**

By (17), a linearization of (1) is of the form

(19) $$\begin{array}{c} y_{n+1}+\frac{2a+b}{a+b+c}y_{n}-\frac{2a+b}{a+b+c}y_{n-1}=0. \end{array}$$

Its characteristic equation is

(20) $$\begin{array}{c} \lambda^{2}+\frac{2a+b}{a+b+c}\lambda -\frac{2a+b}{a+b+c}=0, \end{array}$$

with eigenvalues $\;\lambda_{\pm }=\left(-\left(2a+b\right)\pm \sqrt{D}\right)/2\left(a+b+c\right)$ , where  *D* = (2*a* + *b*)(6*a* + 5*b* + 4*c*)  . It is clear that  *λ*_−_ < 0  and  *λ*_+_ > 0. Now, we prove that  *λ*_+_ ∈ (0,1)  and

(21) λ−{∈(−1,0)for  3a+b−c<0<−1for  3a+b−c>0=−1for  3a+b−c=0.

Namely,

(i)
$\;\lambda_{+}<1\Leftrightarrow -\left(2a+b\right)+\sqrt{D}<2\left(a+b+c\right)\Leftrightarrow \sqrt{D}<4a+3b+2c\Leftrightarrow 4{\left(a+b+c\right)}^{2}>0$ , which is always satisfied;
(ii) also,
(22) λ−<−1⇔−(2a+b)−D<−2(a+b+c)⇔D>b+2c⇔(2a+b)2>(a−c)2⇔(a+b+c)(3a+b−c)>0⇔3a+b−c>0;
(iii)   
*λ*_−_ > −1⇔3*a* + *b* − *c* < 0  and  *λ*_−_ = −1⇔3*a* + *b* − *c* = 0.

Also, if  3*a* + *b* − *c* = 0, then  *c* = 3*a* + *b*, and we have  *λ*_+_ = 1/2.

## 4. Periodic Solutions

In this section, we present results for the existence of minimal period-two solutions of (7).

**Theorem 10**

(a) Equation (1) has the minimal period-two solution

(23) $$\begin{array}{c} \left\{\ldots ,0,\frac{1}{c},0,\frac{1}{c},\ldots \right\}, \end{array}$$

for all positive values of parameters  *a*, *b*, and  *c*.

(b) If 3*a* + *b* − *c* < 0, then (1) has the minimal period-two solution:

(24) $$\begin{array}{c} \left\{\ldots ,\varphi ,\psi ,\varphi ,\psi ,\ldots \right\},\;\left(\varphi \neq \psi ,\;\;\varphi >0,\;\;\psi >0\right), \end{array}$$

where

(25) $$\begin{array}{c} \varphi =\frac{1-\sqrt{D}}{2\left(c-a\right)},\;\;\psi =\frac{1+\sqrt{D}}{2\left(c-a\right)},\;D=\frac{3a+b-c}{b-a-c}>0. \end{array}$$

**Proof**

Suppose that there is a minimal period-two solution  {*φ*, *ψ*, *φ*, *ψ*,…}  of (1), where  *φ*  and  *ψ*  are distinct nonnegative real numbers such that  *φ* ^2^ + *ψ* ^2^ ≠ 0. Then,  *φ*, *ψ*  satisfy

(26) $$\begin{array}{c} \varphi =\frac{\varphi^{2}}{a\psi^{2}+b\psi \varphi +c\varphi^{2}},\\ \psi =\frac{\psi^{2}}{a\varphi^{2}+b\varphi \psi +c\psi^{2}}, \end{array}$$

from which we obtain three cases:

(27) $$\begin{array}{c} \varphi =0,\\ a\varphi^{2}+b\varphi \psi +c\psi^{2}=\psi , \end{array}$$

(28) $$\begin{array}{c} a\psi^{2}+b\varphi \psi +c\varphi^{2}=\varphi ,\\ \psi =0, \end{array}$$

(29) $$\begin{array}{c} a\psi^{2}+b\varphi \psi +c\varphi^{2}=\varphi , \end{array}$$

(30) $$\begin{array}{c} a\varphi^{2}+b\varphi \psi +c\psi^{2}=\psi . \end{array}$$

Conclusion (*a*) follows from (27) and (28).

Subtracting (30) from (29), we have

(31) $$\begin{array}{c} \left(\psi -\varphi \right)\left[\left(a-c\right)\left(\varphi -\psi \right)+1\right]=0, \end{array}$$

that is,

(32) $$\begin{array}{c} \varphi +\psi =\frac{1}{c-a} \end{array}$$

for  *a* < *c*. Substituting (32) in (29), we obtain

(33) $$\begin{array}{c} \varphi^{2}-\frac{1}{c-a}\varphi +\frac{a}{\left(a-b+c\right){\left(c-a\right)}^{2}}=0, \end{array}$$

from which

(34) $$\begin{array}{c} \varphi_{\pm }=\frac{1\pm \sqrt{D}}{2\left(c-a\right)}. \end{array}$$

Equation (32) implies that

(35) $$\begin{array}{c} \psi_{\pm }=\frac{1}{c-a}-\varphi_{\pm }=\varphi_{\mp }. \end{array}$$

It is clear that

(36) D=3a+b−cb−a−c>0⇔{(3a+b−c>0∧b−a−c>0)  ×∨(3a+b−c<0∧b−a−c<0)}⇔(b−a>c∨3a+b<c).

If  *b* − *a* > *c*, then

(37) D=1+4ab−a−c>1⇒(φ−<0, φ+>0)⇒(ψ−>0, ψ+<0),

which is a contradiction.

If  3*a* + *b* < *c*, then

(38) $$\begin{array}{c} 0<D=1+\frac{4a}{b-a-c}<1\Rightarrow \left(\varphi_{\pm }>0,\;\psi_{\pm }>0\right). \end{array}$$

By using substitution

(39) $$\begin{array}{c} x_{n-1}=u_{n},\\ x_{n}=v_{n}, \end{array}$$

equation (1) becomes the system of equations

(40) $$\begin{array}{c} u_{n+1}=v_{n},\\ v_{n+1}=\frac{u_{n}^{2}}{av_{n}^{2}+bu_{n}v_{n}+cu_{n}^{2}}. \end{array}$$

The map  *T*  corresponding to the system (40) is of the form

(41) $$\begin{array}{c} T\left(\begin{array}{c} u\\ v \end{array}\right)=\left(\begin{array}{c} v\\ g\left(u,v\right) \end{array}\right), \end{array}$$

where  *g*(*u*, *v*) = *u* ^2^/(*av* ^2^ + *buv* + *cu* ^2^).  The second iteration of the map  *T*  is

(42) $$\begin{array}{c} T^{2}\left(\begin{array}{c} u\\ v \end{array}\right)=T\left(\begin{array}{c} v\\ g\left(u,v\right) \end{array}\right)=\left(\begin{array}{c} g\left(u,v\right)\\ g\left(v,g\left(u,v\right)\right) \end{array}\right)=\left(\begin{array}{c} F\left(u,v\right)\\ G\left(u,v\right) \end{array}\right), \end{array}$$

where

(43) $$\begin{array}{c} F\left(u,v\right)=g\left(u,v\right),G\left(u,v\right)=\frac{v^{2}}{aF^{2}\left(u,v\right)+bvF\left(u,v\right)+cv^{2}}, \end{array}$$

and the map  *T* ^2^  is competitive by Remark 7. The Jacobian matrix of the map  *T* ^2^  is

(44) $$\begin{array}{c} J_{T^{2}}\left(\begin{array}{c} u\\ v \end{array}\right)=\left(\begin{array}{cc} \frac{\partial F}{\partial u} & \frac{\partial F}{\partial v}\\ \frac{\partial G}{\partial u} & \frac{\partial G}{\partial v} \end{array}\right). \end{array}$$

Notice that periodic solutions  (0, 1/*c*),  (1/*c*, 0),  (*φ*, *ψ*), and  (*ψ*, *φ*)  of (1) are equilibrium points of the map  *T* ^2^.

Now, we have

(45) ∂F∂u=2u(av2+buv+cu2)−u2(bv+2cu)(av2+buv+cu2)2=uv(2av+bu)(av2+buv+cu2)2,∂F∂v=−u2(2av+bu)(av2+buv+cu2)2,∂G∂u=−v2(aF2(u,v)+bvF(u,v)+cv2)2 ·(2aF(u,v)+bv)∂F∂u,∂G∂v=(2v(aF2(u,v)+bvF(u,v)+cv2) −v2(2aF(u,v)(∂F/∂v)+bF(u,v) +bv(∂F/∂v)+2cv))  ×(aF2(u,v)+bvF(u,v)+cv2)−2=(v(2aF(u,v)+bv)F(u,v) −(2aF(u,v)+bv)v2(∂F/∂v))  ×(aF2(u,v)+bvF(u,v)+cv2)−2.

**Theorem 11**

(i) The minimal period-two points

(46) $$\begin{array}{c} \left(0,\frac{1}{c}\right),\;\;\left(\frac{1}{c},0\right) \end{array}$$

are locally asymptotically stable.

(ii) If *a* < *c* and 3*a* + *b* − *c* < 0, then the minimal period-two points

(47) $$\begin{array}{c} \left(\varphi ,\psi \right),\;\;\left(\psi ,\varphi \right)\;\left(\varphi \neq \psi ,\;\;\varphi >0,\;\;\psi >0\right), \end{array}$$

where  *φ*  and  *ψ*  satisfy (25), are saddle points.

**Proof**

(i) Since  *F*(0, 1/*c*) = 0, for periodic point  (0, 1/*c*), we have

(48) $$\begin{array}{c} \frac{\partial F}{\partial u}\left(\begin{array}{c} 0\\ \frac{1}{c} \end{array}\right)=\frac{\partial F}{\partial v}\left(\begin{array}{c} 0\\ \frac{1}{c} \end{array}\right)=\frac{\partial G}{\partial u}\left(\begin{array}{c} 0\\ \frac{1}{c} \end{array}\right)=\frac{\partial G}{\partial v}\left(\begin{array}{c} 0\\ \frac{1}{c} \end{array}\right)=0,\\ J_{T^{2}}\left(\begin{array}{c} 0\\ \frac{1}{c} \end{array}\right)=\left(\begin{array}{cc} 0 & 0\\ 0 & 0 \end{array}\right) \end{array}$$

with eigenvalues  *λ*_1,2_ = 0, which implies that  (0, 1/*c*)  is locally asymptotically stable.

Similarly, since  *F*(1/*c*, 0) = 1/*c*, for periodic solution  (1/*c*, 0), we have

(49) $$\begin{array}{c} J_{T^{2}}\left(\begin{array}{c} \frac{1}{c}\\ 0 \end{array}\right)=\left(\begin{array}{cc} 0 & -\frac{b}{c}\\ 0 & 0 \end{array}\right) \end{array}$$

with eigenvalues  *λ*_1,2_ = 0, which implies that  (0, 1/*c*)  is locally asymptotically stable.

(ii) By (26), we have that

(50) $$\begin{array}{c} F\left(\varphi ,\psi \right)=\varphi , \end{array}$$

(51) $$\begin{array}{c} \frac{\partial F}{\partial u}\left(\begin{array}{c} \varphi \\ \psi \end{array}\right)=\psi \left(2a\frac{\psi }{\varphi }+b\right), \end{array}$$

(52) $$\begin{array}{c} \frac{\partial F}{\partial v}\left(\begin{array}{c} \varphi \\ \psi \end{array}\right)=-\varphi \left(2a\frac{\psi }{\varphi }+b\right). \end{array}$$

By (50) and (51), we obtain

(53) ∂G∂u(φψ)=−ψ2(aφ2+bψφ+cψ2)2 ·(2aφ+bψ)∂F∂u(φψ)=−ψ(2aφ+bψ)(2aψφ+b);

that is,

(54) $$\begin{array}{c} \frac{\partial G}{\partial u}\left(\begin{array}{c} \varphi \\ \psi \end{array}\right)=-\psi^{2}\left(2a\frac{\varphi }{\psi }+b\right)\left(2a\frac{\psi }{\varphi }+b\right). \end{array}$$

Similarly, we have

(55) ∂G∂v(φψ)=ψ(2aφ+bψ)φ−(2aφ+bψ)ψ2(∂F/∂v)(φψ)(aφ2+bψφ+cψ2)2=ψ(2aφ+bψ)φ+(2aφ+bψ)ψ2(2aψ+bφ)ψ2,

so that

(56) $$\begin{array}{c} \frac{\partial G}{\partial v}\left(\begin{array}{c} \varphi \\ \psi \end{array}\right)=\left(2a\frac{\varphi }{\psi }+b\right)\varphi +\left(2a\frac{\varphi }{\psi }+b\right)\left(2a\frac{\psi }{\varphi }+b\right)\varphi \psi . \end{array}$$

Now, we obtain that Jacobian matrix of the map  *T* ^2^  at the point  (*φ*, *ψ*)  is of the form

(57) $$\begin{array}{c} J_{T^{2}}\left(\begin{array}{c} \varphi \\ \psi \end{array}\right)=\left(\begin{array}{cc} \frac{\partial F}{\partial u}\left(\begin{array}{c} \varphi \\ \psi \end{array}\right) & \frac{\partial F}{\partial v}\left(\begin{array}{c} \varphi \\ \psi \end{array}\right)\;\\ \frac{\partial G}{\partial u}\left(\begin{array}{c} \varphi \\ \psi \end{array}\right) & \frac{\partial G}{\partial v}\left(\begin{array}{c} \varphi \\ \psi \end{array}\right) \end{array}\right). \end{array}$$

The corresponding characteristic equation is

(58) $$\begin{array}{c} \lambda^{2}-p\lambda +q=0, \end{array}$$

where

(59) p=∂F∂u(φψ)+∂G∂v(φψ)=ψ(2aψφ+b)+φ(2aφψ+bψ) +φψ(2aφψ+b)(2aψφ+b),

that is,

(60) $$\begin{array}{c} p=m+n+mn, \end{array}$$

where

(61) $$\begin{array}{c} m=\psi \left(2a\frac{\psi }{\varphi }+b\right),\;\;n=\varphi \left(2a\frac{\varphi }{\psi }+b\right). \end{array}$$

Notice that

(62) $$\begin{array}{c} \frac{\partial F}{\partial u}\left(\begin{array}{c} \varphi \\ \psi \end{array}\right)=m,\;\;\;\frac{\partial F}{\partial v}\left(\begin{array}{c} \varphi \\ \psi \end{array}\right)=-\frac{\varphi }{\psi }m,\\ \frac{\partial G}{\partial u}\left(\begin{array}{c} \varphi \\ \psi \end{array}\right)=-\frac{\psi }{\varphi }mn,\;\;\;\frac{\partial G}{\partial v}\left(\begin{array}{c} \varphi \\ \psi \end{array}\right)=n\left(1+m\right), \end{array}$$

so that

(63) $$\begin{array}{c} q=\det J_{T^{2}}\left(\begin{array}{c} \varphi \\ \psi \end{array}\right)=mn\left(1+m\right)-m^{2}n=mn. \end{array}$$

We need to show that

(64) $$\begin{array}{c} \left|p\right|>\left|1+q\right|\;\;p^{2}-4q>0. \end{array}$$

(i) Consider that
(65) p2−4q>0⇔(m+n+mn)2−4mn>0⇔m2+n2+m2n2+2m2n+2mn2>2mn,
  which is satisfied because  *m* ^2^ + *n* ^2^ ≥ 2*mn*.  
(ii) Notice that
(66) m=ψ(2aψφ+b)=2aψ2φ+bψ=(26)2φ(φ−bφψ−cφ2)+bψ=2−bψ−2cφn=φ(2aφψ+b)=2aφ2ψ+bφ=(26)2ψ(ψ−bφψ−cψ2)+bφ=2−bφ−2cφ.

This implies that

(67) |p|>|1+q|⇔p>1+q⇔m+n>1⇔3−(b+c)(φ+ψ)>0⇔(18)3−b+cc−a>0⇔3a+b−c<0,

which is satisfied.

## 5. Global Results and Basins of Attraction

In this section, we present global dynamics results for (1).

Notice that  *T*(*u*, 0) = (0, 1/*c*),  *u* > 0, and  *T*(0, *v*) = (*v*, 0),  *v* > 0.

**Theorem 12**

If 3*a* + *b* − *c* < 0, then (7) has a unique equilibrium point $\bar{x}$ , which is locally asymptotically stable, and has the minimal period-two solution (*φ*, *ψ*), (*ψ*, *φ*), which is a saddle point and has the minimal period-two solution (0, 1/*c*), (1/*c*, 0) which is locally asymptotically stable. The basin of attraction $ℬ\left(\left(\bar{x},\bar{x}\right)\right)$ of $\left(\bar{x},\bar{x}\right)$ is the region between the global stable sets *𝒲* ^*s*^((*φ*, *ψ*)) and *𝒲* ^*s*^((*ψ*, *φ*)). The basins of attraction *ℬ*((*φ*, *ψ*)) = *𝒲* ^*s*^((*φ*, *ψ*)) and *ℬ*((*ψ*, *φ*)) = *𝒲* ^*s*^((*ψ*, *φ*)) are exactly the global stable sets of (*φ*, *ψ*) and (*ψ*, *φ*). Furthermore, the basin of attraction of the minimal period-two solution (0, 1/*c*), (1/*c*, 0) is the union of the regions above *𝒲* ^*s*^((*φ*, *ψ*)) and below *𝒲* ^*s*^((*ψ*, *φ*)) in SE ordering; that is,

- if  (*x*_−1_, *x*_0_) ∈ *𝒲*_−_((*φ*, *ψ*)), then  lim⁡_*n*→*∞*_ *x*_2*n*_ = 1/*c*  and  lim⁡_*n*→*∞*_ *x*_2*n*+1_ = 0;
- if  (*x*_−1_, *x*_0_) ∈ *𝒲*_+_(*ψ*, *φ*), then  lim⁡_*n*→*∞*_ *x*_2*n*_ = 0  and  lim⁡_*n*→*∞*_ *x*_2*n*+1_ = 1/*c*.

**Proof**

Using assumption  3*a* + *b* − *c* < 0  (and its consequences:  *c* − *a* > 0, *b* − *c* − *a* < 0) and (25), it is easy to check that $\;\left(0,1/c\right)\;{⪯}_{\text{se}}\;\left(\varphi ,\psi \right)\;{⪯}_{\text{se}}\;\left(\bar{x},\bar{x}\right)\;{⪯}_{\text{se}}\;\left(\psi ,\varphi \right)\;{⪯}_{\text{se}}\;\left(1/c,0\right)$ . It is easy to check that the equilibrium point $\;\left(\bar{x},\bar{x}\right)\;$ is locally asymptotically stable for the strictly competitive map  *T* ^2^  as well. Equation (1) is equivalent to the system of difference equations (40) which can be decomposed into the system of the even-indexed and odd-indexed terms as follows:

(68) $$\begin{array}{c} u_{2n}=v_{2n-1},\\ u_{2n+1}=v_{2n},\\ v_{2n}=\frac{v_{2n-1}^{2}}{au_{2n-1}^{2}+bu_{2n-1}v_{2n-1}+cv_{2n-1}^{2}},\\ v_{2n+1}=\frac{v_{2n}^{2}}{au_{2n}^{2}+bu_{2n}v_{2n}+cv_{2n}^{2}}. \end{array}$$

The conclusion follows from Lemma 9 and from Theorems 10, 11, and 8 and using the facts that

(i) if  (*x*_−1_, *x*_0_) ∈ *𝒲*_−_((*φ*, *ψ*)), then
(69) $$\begin{array}{c} \left(u_{2n},v_{2n}\right)=T^{2n}\left(\left(u_{0},v_{0}\right)\right)\to \left(0,\frac{1}{c}\right),\\ \left(u_{2n+1},v_{2n+1}\right)=T^{2n+1}\left(\left(u_{0},v_{0}\right)\right)\to \left(\frac{1}{c},0\right); \end{array}$$
(ii) if $\;\left(u_{0},v_{0}\right)\in {𝒲}_{+}\left(\left(\bar{x},\bar{x}\right)\right)$ , then
(70) $$\begin{array}{c} \left(u_{2n},v_{2n}\right)=T^{2n}\left(\left(u_{0},v_{0}\right)\right)\to \left(\frac{1}{c},0\right),\\ \left(u_{2n+1},v_{2n+1}\right)=T^{2n+1}\left(\left(u_{0},v_{0}\right)\right)\to \left(0,\frac{1}{c}\right). \end{array}$$

It means that

(i) if $\;\left(x_{-1},x_{0}\right)\in {𝒲}_{-}\left(\left(\bar{x},\bar{x}\right)\right)$ , then  *T* ^2*n*^((*x*_−1_, *x*_0_)) → (0, 1/*c*)  and  *T* ^2*n*+1^((*x*_−1_, *x*_0_)) → (1/*c*, 0), that is,
(71) $$\begin{array}{c} \underset{n\to \infty }{\lim}x_{2n}=\frac{1}{c}\;\;\underset{n\to \infty }{\lim}x_{2n+1}=0; \end{array}$$
(ii) if  (*x*_−1_, *x*_0_) ∈ *𝒲*_+_(*ψ*, *φ*), then  *T* ^2*n*+1^((*x*_−1_, *x*_0_)) → (0, 1/*c*)  and  *T* ^2*n*^((*x*_−1_, *x*_0_)) → (1/*c*, 0), that is,
(72) $$\begin{array}{c} \underset{n\to \infty }{\lim}x_{2n}=0\;\;\underset{n\to \infty }{\lim}x_{2n+1}=\frac{1}{c} \end{array}$$

(see Figure 1).

**Theorem 13**

If 3*a* + *b* − *c* > 0, then (1) has a unique equilibrium point $\bar{x}$ which is saddle point and has the minimal period-two solution (0, 1/*c*), (1/*c*, 0) which is locally asymptotically stable. There exists a set *𝒞* which is an invariant subset of the basin of attraction of $\left(\bar{x},\bar{x}\right)$ . The set *𝒞* is a graph of a strictly increasing continuous function of the first variable on an interval and separates *ℛ*∖(0,0), where *ℛ* = [0, *∞*) × [0, *∞*), into two connected and invariant components ${𝒲}_{-}\left(\left(\bar{x},\bar{x}\right)\right)$ and ${𝒲}_{+}\left(\left(\bar{x},\bar{x}\right)\right)$ which satisfy that

- if $\;\left(x_{-1},x_{0}\right)\in {𝒲}_{-}\left(\left(\bar{x},\bar{x}\right)\right)$ , then  lim⁡_*n*→*∞*_ *x*_2*n*_ = 1/*c*  and  lim⁡_*n*→*∞*_ *x*_2*n*+1_ = 0;
- if $\;\left(x_{-1},x_{0}\right)\in {𝒲}_{+}\left(\left(\bar{x},\bar{x}\right)\right)$ , then  lim⁡_*n*→*∞*_ *x*_2*n*_ = 0  and  lim⁡_*n*→*∞*_ *x*_2*n*+1_ = 1/*c*.

**Proof**

It is easy to check that $\;\left(\bar{x},\bar{x}\right)\;$ is a saddle point for the strictly competitive map  *T* ^2^  as well. The existence of the set *𝒞* with stated properties follows from Lemma 9 and Theorems 2, 4, and 10. Therefore, using (68), we obtain that

(i) if $\;\left(u_{0},v_{0}\right)\in {𝒲}_{-}\left(\left(\bar{x},\bar{x}\right)\right),$ then
(73) $$\begin{array}{c} \left(u_{2n},v_{2n}\right)=T^{2n}\left(\left(u_{0},v_{0}\right)\right)\to \left(0,\frac{1}{c}\right),\\ \left(u_{2n+1},v_{2n+1}\right)=T^{2n+1}\left(\left(u_{0},v_{0}\right)\right)\to \left(\frac{1}{c},0\right); \end{array}$$
(ii) if $\;\left(u_{0},v_{0}\right)\in {𝒲}_{+}\left(\left(\bar{x},\bar{x}\right)\right)$ , then
(74) $$\begin{array}{c} \left(u_{2n},v_{2n}\right)=T^{2n}\left(\left(u_{0},v_{0}\right)\right)\to \left(\frac{1}{c},0\right),\\ \left(u_{2n+1},v_{2n+1}\right)=T^{2n+1}\left(\left(u_{0},v_{0}\right)\right)\to \left(0,\frac{1}{c}\right). \end{array}$$

Consequently,

(i) if $\;\left(x_{-1},x_{0}\right)\in {𝒲}_{-}\left(\left(\bar{x},\bar{x}\right)\right)$ , then  *T* ^2*n*^((*x*_−1_, *x*_0_)) → (0, 1/*c*)  and  *T* ^2*n*+1^((*x*_−1_, *x*_0_)) → (1/*c*, 0); that is,
(75) $$\begin{array}{c} \underset{n\to \infty }{\lim}x_{2n}=\frac{1}{c}\;\;\underset{n\to \infty }{\lim}x_{2n+1}=0; \end{array}$$
(ii) if $\;\left(x_{-1},x_{0}\right)\in {𝒲}_{+}\left(\left(\bar{x},\bar{x}\right)\right)$ , then  *T* ^2*n*+1^((*x*_−1_, *x*_0_)) → (0, 1/*c*)  and  *T* ^2*n*^((*x*_−1_, *x*_0_)) → (1/*c*, 0); that is,
(76) $$\begin{array}{c} \underset{n\to \infty }{\lim}x_{2n}=0\;\;\underset{n\to \infty }{\lim}x_{2n+1}=\frac{1}{c} \end{array}$$

(see Figure 2).

**Theorem 14**

If 3*a* + *b* − *c* = 0, then (1) has a unique equilibrium point $\bar{x}$ which is nonhyperbolic and has two minimal period-two points (0, 1/*c*), (1/*c*, 0) which are locally asymptotically stable points. There exists a set *𝒞* which is an invariant subset of the basin of attraction of $\left(\bar{x},\bar{x}\right)$ . The set *𝒞* is a graph of a strictly increasing continuous function of the first variable on an interval and separates *ℛ*∖(0,0), where *ℛ* = [0, *∞*) × [0, *∞*), into two connected and invariant components ${𝒲}_{-}\left(\left(\bar{x},\bar{x}\right)\right)$ and ${𝒲}_{+}\left(\left(\bar{x},\bar{x}\right)\right)$ which satisfy that

- if $\;\left(x_{-1},x_{0}\right)\in {𝒲}_{-}\left(\left(\bar{x},\bar{x}\right)\right)$ , then  lim⁡_*n*→*∞*_ *x*_2*n*_ = 1/*c*  and  lim⁡_*n*→*∞*_ *x*_2*n*+1_ = 0;
- if $\;\left(x_{-1},x_{0}\right)\in {𝒲}_{+}\left(\left(\bar{x},\bar{x}\right)\right)$ ,  lim⁡_*n*→*∞*_ *x*_2*n*_ = 0  and  lim⁡_*n*→*∞*_ *x*_2*n*+1_ = 1/*c*.

**Proof**

In view of Lemma 9, the eigenvalues of the map  *T*  at the equilibrium point $\;(\bar{x},\bar{x})\;$ are  *λ*_1_ = −1  and  *λ*_2_ = 1/2, which means that  *μ*_1_ = *λ*_1_^2^ = 1  and  *μ*_2_ = *λ*_2_^2^ = 1/4  are the eigenvalues of the map  *T* ^2^.  Using (51), (52), (54), and (56), we obtain

(77) $$\begin{array}{c} J_{T^{2}}\left(\bar{x},\bar{x}\right)=\left(\begin{array}{cc} Q & -Q\\ -Q^{2} & \left(Q-1\right)\left(Q-2\right) \end{array}\right), \end{array}$$

where  *Q* = (2*a* + *b*)/(*a* + *b* + *c*).  A straightforward calculation yields that the eigenvector corresponding to the eigenvalue  *μ*_2_ = 1/4  is of the form

(78) $$\begin{array}{c} v_{2}=\left(\begin{array}{c} 2\alpha \\ \alpha \end{array}\right),\;\alpha \in \mathbb{R}\setminus \left\{0\right\}. \end{array}$$

We see that eigenvector  **v**_2_  is not parallel to coordinate axes. Therefore, all conditions of Theorem 2 are satisfied for the map  *T* ^2^  with *ℛ* = (0, *∞*) × (0, *∞*). As a consequence of this and using (68), we have that

- if $\;\left(u_{0},v_{0}\right)\in {𝒲}_{-}\left(\left(\bar{x},\bar{x}\right)\right),$ then  (*u*_2*n*_, *v*_2*n*_) → (0, 1/*c*)  and  (*u*_2*n*+1_, *v*_2*n*+1_) → (1/*c*, 0);
- if $\;\left(u_{0},v_{0}\right)\in {𝒲}_{+}\left(\left(\bar{x},\bar{x}\right)\right)$ , then  (*u*_2*n*_, *v*_2*n*_) → (1/*c*, 0)  and  (*u*_2*n*+1_, *v*_2*n*+1_) → (0, 1/*c*).

It means that

(i) if $\;\left(x_{-1},x_{0}\right)\in {𝒲}_{-}\left(\left(\bar{x},\bar{x}\right)\right)$ , then  *T* ^2*n*^((*x*_−1_, *x*_0_)) → (0, 1/*c*)  and  *T* ^2*n*+1^((*x*_−1_, *x*_0_)) → (1/*c*, 0); that is,
(79) $$\begin{array}{c} \underset{n\to \infty }{\lim}x_{2n}=\frac{1}{c}\;\;\underset{n\to \infty }{\lim}x_{2n+1}=0; \end{array}$$
(ii) if $\;\left(x_{-1},x_{0}\right)\in {𝒲}_{+}\left(\left(\bar{x},\bar{x}\right)\right)$ , then  *T* ^2*n*+1^((*x*_−1_, *x*_0_)) → (0, 1/*c*)  and  *T* ^2*n*^((*x*_−1_, *x*_0_)) → (1/*c*, 0); that is,
(80) $$\begin{array}{c} \underset{n\to \infty }{\lim}x_{2n}=0\;\;\underset{n\to \infty }{\lim}x_{2n+1}=\frac{1}{c} \end{array}$$

(see Figure 3).

**Remark 15**

As one may notice from the figures all stable manifolds of either saddle point equilibrium points or saddle period-two solutions are asymptotic to the origin, which is the point where (1) is not defined. These manifolds cannot end in any other point on the axes since the union of axes without the origin is an invariant set. Thus, the limiting points of the global stable manifolds of either saddle point equilibrium points or saddle period-two solutions have endpoints at  (0,0)  and  (*∞*, *∞*).

## Figures and Tables

**Figure 1:**
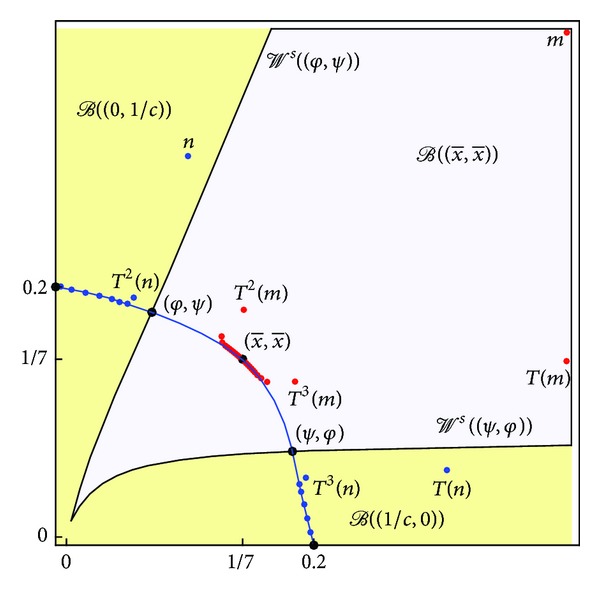
Global dynamics of (1) for  *a* = 1,  *b* = 1, and  *c* = 5—an illustration of Theorem 12.

**Figure 2:**
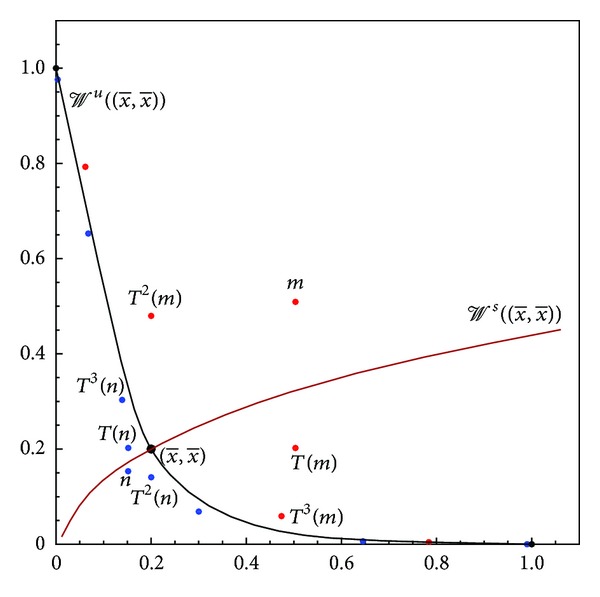
Global dynamics of (1) for  *a* = 2,  *b* = 2, and  *c* = 1—an illustration of Theorem 13.

**Figure 3:**
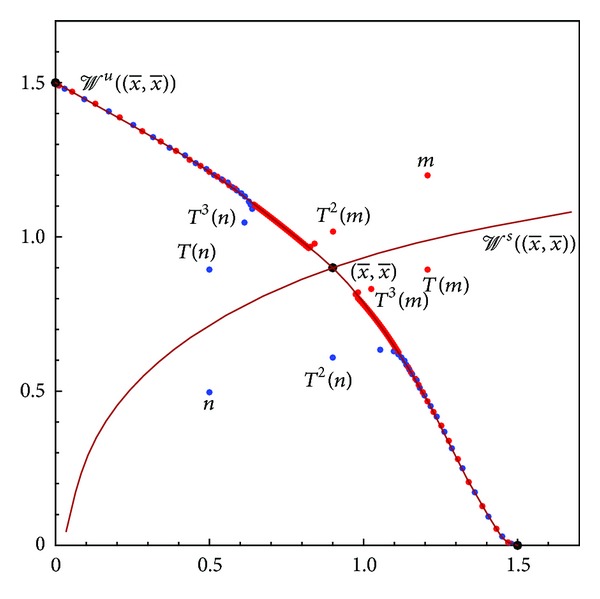
Global dynamics of (1) for  *a* = 1/9,  *b* = 1/3, and  *c* = 2/3—an illustration of Theorem 14.
